# Investigating the association factors of acute postpartum pain: a cohort study

**DOI:** 10.1186/s12871-023-02214-w

**Published:** 2023-07-25

**Authors:** Chin Wen Tan, Nicole Y-Kit Tan, Rehena Sultana, Hon Sen Tan, Ban Leong Sng

**Affiliations:** 1 Department of Women’s Anesthesia, KK Women’s and Children’s Hospital, 100 Bukit Timah Road, 229899 Singapore, Singapore; 2 Anesthesiology and Perioperative Sciences Academic Clinical Program, Duke-NUS Medical School, 8 College Road, Singapore, Singapore; 3 Duke-NUS Medical School, 8 College Road, Singapore, Singapore; 4 Center for Quantitative Medicine, Duke-NUS Medical School, 8 College Road, Singapore, Singapore

**Keywords:** Labor pain, Postpartum pain, Labor analgesia

## Abstract

**Background:**

Labor pain intensity is known to predict persistent postpartum pain, whereas acute postpartum pain may interfere with maternal postpartum physical, mental, and emotional well-being. Nevertheless, there is little research studying the association between labor pain intensity and acute postpartum pain. This study investigated the associations between labor pain intensity and psychological factors with acute postpartum pain.

**Methods:**

We included women with American Society of Anesthesiologists (ASA) physical status II, having ≥ 36 gestational weeks and a singleton pregnancy. We investigated the association between labor pain intensity (primary exposure) and high acute postpartum pain at 0 to 24 h after delivery (Numeric Rating Scale (NRS) ≥ 3 of 10; primary outcome). Pre-delivery questionnaires including Angle Labor Pain Questionnaire (A-LPQ), Pain Catastrophizing Scale (PCS), Fear Avoidance Components Scale (FACS) and State Trait Anxiety Inventory (STAI) were administered. Demographic, pain, obstetric and neonatal characteristics were also collected accordingly.

**Results:**

Of the 880 women studied, 121 (13.8%) had high acute postpartum pain at 0 to 24 h after delivery. A-LPQ total, PCS, FACS and STAI scores were not significantly associated with acute postpartum pain. Greater A-LPQ subscale on birthing pain (adjusted odds ratio (aOR) 1.03, 95% CI 1.01–1.05, p = 0.0008), increased blood loss during delivery (for every 10ml change; aOR 1.01, 95% CI 1.00–1.03, p = 0.0148), presence of shoulder dystocia (aOR 10.06, 95% CI 2.28–44.36, p = 0.0023), and use of pethidine for labor analgesia (aOR 1.74, 95% CI 1.07–2.84, p = 0.0271) were independently associated with high acute postpartum pain. “Sometimes” having nausea during menstruation before current pregnancy (aOR 0.34, 95% CI 0.16–0.72, p = 0.0045) was found to be independently associated with reduced risk of high acute postpartum pain.

**Conclusions:**

Pre-delivery pain factor together with obstetric complications (shoulder dystocia, blood loss during delivery) were independently associated with high acute postpartum pain.

**Trial registration:**

This study was registered on clinicaltrials.gov registry (NCT03167905) on 30/05/2017.

## Introduction

Despite being a natural process, labor and delivery may turn out to be a source of major trauma to the mother mentally and physically. It is reported that around 60% of nulliparous and 45% of multiparous women may experience severe pain during the first stage of labor [1]. Epidural analgesia is considered the gold standard to relieve labor pain, however some patients may have contraindications to this modality, limited or no access to its use, or opt for analgesia of lesser invasiveness. In addition, pain experiences during labor may differ among individuals and pregnancies, and is influenced by a multitude of physiological and psychosocial factors [1, 2]. Previous studies reported that psychological factors such as greater pain catastrophizing, anxiety sensitivity, and fear avoidance are associated with increased labor pain at early stage, and could lead to longer postpartum recovery [3–5]. Additionally, labor pain intensity in vaginal delivery is known to predict persistent postpartum pain [6]. Thus, a comprehensive multidimensional measurement of labor pain is critical for patient care and management to prevent long-term maternal morbidity.

Conventional pain measures (e.g., Visual Analog Scale (VAS), Numeric Rating Scale (NRS)) encompass a unidimensional assessment on pain intensity without considering other pain perception and experience. To address this issue, Angle et al. developed Angle Labor Pain Questionnaire (A-LPQ) that provides a comprehensive multidimensional scale that includes the psychological status, locations, and perception of labor pain that is specific to the context of labor and delivery [7]. A-LPQ is demonstrated to have good internal consistency and test-retest probability for both total and subscale scores, and is positively correlated with NRS and Verbal Rating Scale (VRS) scores [7]. A-LPQ is also linguistically validated, suitable for use among local population in Singapore [8].

Effective identification and management of acute postpartum pain is crucial in determining maternal outcomes and satisfaction. Eisenach et al. reported that severe acute postpartum pain is significantly associated with a 2.5-fold increase in risk of persistent postpartum pain and 3-fold increase in risk of postpartum depression [9]. Studies have also investigated the association between psychological factors (e.g., pain catastrophizing, fear avoidance, anxiety), but their association with acute postpartum pain is less known [3, 10, 11]. Furthermore, our previous data in nulliparous laboring women showed that those who had sub-acute pain after childbirth (SAPC; postpartum pain that lasts between four weeks to three months) was associated with receiving analgesia prior to neuraxial procedure (nitrous oxide/ meperidine), prolonged neuraxial procedure and multiple attempts, maternal anxiety and stress, and obstetric complications (e.g., blood loss during delivery, emergency cesarean delivery); yet their association with acute postpartum pain is unclear [12].

Despite the fact that both labor pain intensity and acute postpartum pain are reported to be associated with persistent postpartum pain, currently the association between these two factors has yet to be established. Exploring and understanding this association may improve peripartum pain management, leading to better patient outcomes. The primary aim of this study was to investigate the association between the labor pain intensity (measured via A-LPQ) and high acute postpartum pain at 0 to 24 h after delivery (measured via NRS ≥ 3 of 10). We would also evaluate the associations of other psychological and pain factors with high acute postpartum pain.

## Methods

### Patient recruitment

The patients recruited in this study were originally consented to participate in a randomized controlled trial on evaluating the association between labor epidural analgesia and postpartum depression (primary study), with the outcome being the incidence of postpartum depression at 6–10 weeks after delivery. Patient recruitment and follow-up for the primary study has been completed with ongoing data analysis. This study is the secondary analysis by using the trial as a platform to investigate the pain components, of which the pain outcomes were not relevant to the primary objective of the trial. The study was reviewed and approved by SingHealth Centralized Institutional Review Board (Ref no. 2017/2090) on 25/03/2017, with registration on Clinicaltrials.gov (NCT03167905) on 30/05/2017. All patients were recruited from June 2017 to July 2021 with written informed consent obtained from all participants on the primary trial, and patients were informed that the data obtained would be used for further analysis such as this study. This manuscript adheres to the Strengthening the Reporting of Observational studies in Epidemiology (STROBE) guidelines.

The study population included women with American Society of Anesthesiologists (ASA) physical status II [13], having ≥ 36 gestational weeks and a singleton pregnancy. Women who had multiple pregnancies, with non-cephalic fetal presentation, presence of obstetric and uncontrolled medical complications, or underwent elective cesarean delivery were excluded from the study. As this study involved the use of validated English questionnaires, participants who could not understand nor read English were also excluded.

### Questionnaires and other collected data

A-LPQ is a 22-item multidimensional questionnaire assessing five subscales on labor pain experience: Uterine contraction pain, fear/ anxiety, back pain/ long haul, birthing pain, and the enormity of pain [7]. The questions are rated from a scale of 0 being none to 10 being worse possible or extremely. Subscale and total scores were calculated accordingly as continuous variable, with higher A-LPQ scores implying a higher labor pain. During the study, patients would receive A-LPQ questionnaire in a private ward setting prior to their labor process, and they were instructed to fill in the questionnaire once they experienced labor pain. Apart from A-LPQ, patients were also administered questionnaires on their psychological characteristics including:

(i) Pain Catastrophizing Scale (PCS): A 13-item scale that measures one’s tendency of developing pain through negative thinking. The instrument comprises three subscales: rumination, magnification, and helplessness and are assessed on a 5-point Likert scale [14]. In this study, apart from studying the total and subscales scores as continuous variables, a cut-off score of 25 would also be used to categorize the patients into high and low pain catastrophizers groups [15]. This cut-off was adopted from a local study that investigated and showed the significant association between pre-delivery pain catastrophizing and increased risk of postpartum depression at 5 to 9 weeks after delivery [15].
(ii) Fear-Avoidance Components Scale (FACS): An instrument to assess one’s fear avoidance associated with painful stimuli [16]. This questionnaire consists of 20 items on a 6-point Likert-like scale from zero (completely disagree) to five (completely agree). Higher FACS scores may indicate greater fear-avoidance of labor pain.
(iii) State Trait Anxiety Inventory (STAI): A 40-item questionnaire that measures state anxiety (current anxiety state) and trait anxiety (susceptibility to perceive anxiety across many situations) [17].

Data on demographic characteristics (age, race, marital status, occupation, etc.) were also collected accordingly. After their delivery, patients were assessed on their pain as part of the routine practice. Data on acute postpartum pain (at rest) at 0 to 24 h were retrieved from electronic record, and was defined as NRS 0 being no pain to 10 being worse pain imaginable. Other data including pain (labor analgesia used), obstetric (mode of delivery, postpartum complications, blood loss, shoulder dystocia) and neonatal (infant weight and length, APGAR scores) characteristics were also recorded.

### Statistical analysis

The primary objective was the association between labor pain intensity and acute postpartum pain at 0 to 24 h after delivery. The primary exposure of labor pain intensity (A-LPQ total and subscale scores) would be treated as continuous variable; whereas the primary outcome of acute postpartum pain would be in binary form: “high acute postpartum pain” for those who scored NRS ≥ 3 of 10 at 0 to 24 h after delivery, and “low acute postpartum pain” for those with NRS < 3 of 10. A NRS cut-off of 3 is considered clinically relevant as patients who score 3 and above would require medical intervention to relieve pain as per clinical practice, including our institution [18]. All demographic, pain, obstetric and neonatal characteristics were presented based on their acute postpartum pain status. Categorical and continuous variables were presented as frequency (proportion) and mean (standard deviation (SD)) respectively. Univariate and multivariable logistic regression were used to determine the potential factors associated with high acute postpartum pain. The quantitative associations derived from the logistic regression models were expressed as odds ratio (OR) with corresponding 95% confidence interval (95% CI). Variables with p < 0.15 with less than 10% of missing data in the univariate logistic regression analysis and clinically relevant were chosen for the subsequent multivariable logistic regression analysis [15, 19]. Stepwise variable selection method was used to finalize the final multivariable model. Statistical significance was set at p < 0.05 and all tests were two-tailed. All analyses were performed using SAS version 9.4 software (SAS Institute, Cary, North Carolina, USA).

## Results

**Fig. 1:**
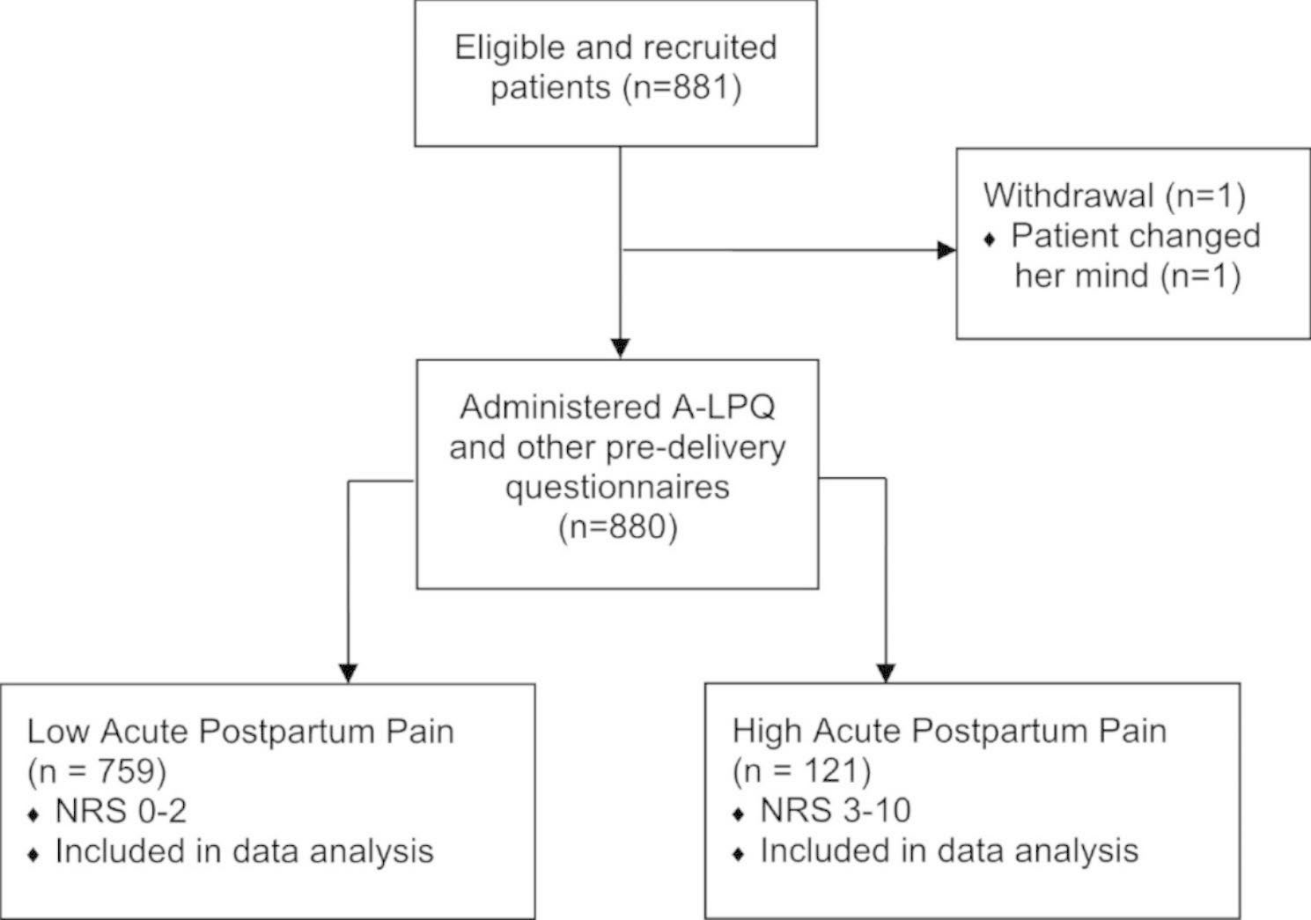
Study flow diagram. *A-LPQ* Angle-Labor Pain Questionnaire; *NRS* Numeric Rating Scale

All patients from the primary study (n = 881) were included for this secondary analysis, and only one patient was excluded from the study before the administration of the questionnaires as she changed her mind to participate in the study (Fig. 1). Among the 880 who completed the pre-delivery questionnaires, 121 patients (13.8%) reported having NRS ≥ 3 (“high acute postpartum pain” group) and 759 (86.3%) reported having NRS less than 3 (“low acute postpartum pain”). There is no significant difference in demographic characteristics in both groups except housing status, whereby living in a rented house (OR 1.84, 95% CI 1.04–3.27, p = 0.0368) was significantly associated with high acute postpartum pain in univariate analysis (Table 1).

**Table 1:** Demographic characteristics

Variables	Low acute postpartum pain (NRS 0–2) N = 759	High acute postpartum pain (NRS ≥ 3) N = 121	Unadjusted OR (95% CI)	P-value
Age (years), mean (SD)	30.9 (4)	30.9 (3.9)	0.99 (0.95–1.04)	0.8197
Race, *n* (%)				0.9576 ^a^
Chinese	402 (53.5)	65 (55.1)	Reference	
Malay	211 (28.1)	31 (26.3)	0.87 (0.38–1.99)	0.7347
Indian	50 (6.6)	7 (5.9)	0.91 (0.57–1.44)	0.6825
Others	89 (11.8)	15 (12.7)	1.04 (0.57–1.91)	0.8934
Marital status, n (%)				0.3959 ^a^
Married	724 (96.8)	117 (100)	Reference	--
Single/ divorced/ separated	24 (3.2)	0	0.14 (0.01–2.42)	0.1750
Weight (kg), mean (SD)	73.3 (13.3)	74.1 (14.4)	1.00 (0.99–1.02)	0.6198
BMI (kg/ m^2^), mean (SD)	29.2 (9.6)	28.5 (4.9)	0.99 (0.94–1.03)	0.5059
Housing type, n (%)				
Public	586 (89.3)	97 (93.3)	Reference	--
Private	70 (10.7)	7 (6.7)	0.60 (0.27–1.35)	0.2204
Housing status, n (%)				
Owned	519 (88.9)	78 (81.3)	Reference	--
Renting	65 (11.1)	18 (18.8)	1.84 (1.04–3.27)	0.0368
Occupation, n (%)				0.7568^a^
Homemaker/ unemployed self-employed/	123 (17.0)	16 (14.7)	Reference	--
Professional/ management	349 (48.3)	52 (47.7)	1.15 (0.63–2.08)	0.6558
Service/ sales/ others	250 (34.6)	41 (37.6)	1.26 (0.68–2.34)	0.4616
Highest educational status, n (%)				
Post-secondary and below	602 (85.3)	99 (87.6)	Reference	--
Post-graduate	104 (14.7)	14 (12.4)	0.82 (0.45–1.49)	0.5112
Personal history of psychiatric illness, n (%) ^b^				
No	704 (92.8)	113 (93.4)	Reference	--
Yes	55 (7.2)	8 (6.6)	0.91 (0.42–1.95)	0.8015
Family history of psychiatric illness, n (%) ^c^				
No	701 (92.4)	108 (89.3)	Reference	--
Yes	58 (7.6)	13 (10.7)	1.45 (0.77–2.74)	0.2470

P values are based on fisher’s exact test for categorical variable and two sample t-test or Mann-Whitney U - test for continuous variables. The missing data are as follows: race (n = 10), marital status (n = 15), housing type (n = 120), housing status (n = 200), occupation (n = 49), and highest education level (n = 61)

*BMI* body mass index; *CI* confidence interval; *NRS* numerical rating scale; *OR* odds ratio; *SD* standard deviation

^a^ Type III p-value

^b^ The subcategories of those with a personal history of psychiatric illness were: depression (n = 22), other mood disorders (n = 1), and others (n = 43)

^c^ The subcategories of those with a family history of psychiatric illness were: depression (n = 20), bipolar (n = 7), other mood disorders (n = 2), and others (n = 45)

We examined the association between pre-delivery psychological measures with acute postpartum pain at 0 to 24 h after delivery (Table 2). Looking into the labor pain intensity, women with high acute postpartum pain had a mean (SD) A-LPQ total score of 130.8 (46.4), whereas those with low acute postpartum pain had a mean (SD) A-LPQ total score of 127.2 (50.2). However, no significant association was found between A-LPQ total score and acute postpartum pain (OR 1.00, 95% CI 1.00–1.01, p = 0.4738). Interestingly, A-LPQ subscale of birthing pain showed a significant association with high acute postpartum pain in univariate analysis (OR 1.03, 95% CI 1.01–1.04, p = 0.0022), and no other A-LPQ subscales demonstrated significant difference among the low and high acute postpartum pain groups. A total of 275 women were high catastrophizers (PCS ≥ 25), of whom 30.2% had high acute postpartum pain and 32.3% had low acute postpartum pain. Nevertheless, PCS total and subscale scores, together with FACS and STAI, did not show significant association with acute postpartum pain.

**Table 2:** Psychological characteristics

Pre-delivery questionnaires	Low acute postpartum pain (NRS 0–2) N = 759	High acute postpartum pain (NRS ≥ 3) N = 121	Unadjusted OR (95% CI)	P-value
A-LPQ, mean (SD)				
Uterine contraction pain subscale (0 to 40)	25.7 (10.6)	25.6 (9.1)	1.00 (0.98–1.02)	0.9088
Fear/ anxiety subscale (0 to 40)	22.7 (11.0)	23.0 (10.1)	1.00 (0.99–1.02)	0.7646
Back pain/ long haul subscale (0 to 50)	30.1 (14.4)	30.6 (13.8)	1.00 (0.99–1.02)	0.7158
Birthing pain subscale (0 to 40)	21.0 (13.6)	25.2 (12.0)	1.03 (1.01–1.04)	0.0022
The enormity of pain subscale (0 to 50)	27.5 (15.5)	27.4 (15.2)	1.00 (0.99–1.01)	0.9796
Total score (0 to 220)	127.2 (50.2)	130.8 (46.4)	1.00 (1.00–1.01)	0.4738
PCS, mean (SD)				
Helplessness subscale (0 to 24)	7.6 (5.3)	7.3 (5.4)	0.99 (0.96–1.03)	0.5945
Magnification subscale (0 to 12)	4.1 (2.8)	4.0 (2.6)	1.00 (0.93–1.07)	0.8948
Rumination subscale (0 to 16)	7.1 (4.4)	7.0 (4.3)	0.99 (0.95–1.04)	0.7449
Total score (0 to 52)	18.8 (11.4)	18.3 (11.3)	1.00 (0.98–1.01)	0.6946
PCS total score, n (%)				
No (PCS < 25)	503 (67.7)	81 (69.8)	Reference	--
Yes (PCS ≥ 25)	240 (32.3)	35 (30.2)	0.91 (0.59–1.39)	0.6477
STAI, mean (SD)				
State anxiety (20 to 80)	39.4 (11.4)	40.1 (10.5)	1.01 (0.99–1.02)	0.5303
Trait anxiety (20 to 80)	38.6 (9.4)	38.0 (8.9)	1.00 (0.98–1.02)	0.5462
Total anxiety (40 to 160)	78.0 (19.2)	78.1 (17.6)	1.00 (0.99–1.01)	0.9398
FACS (0 to 100), mean (SD)	37.7 (18.4)	38.7 (17.4)	1.00 (0.99–1.01)	0.5973

*A-LPQ* Angle Labor Pain Questionnaire; *CI* confidence interval; *FACS* Fear Avoidance Components Scale; *NRS* numerical rating scale; *OR* odds ratio; *PCS* Pain Catastrophizing Scale; *SD* standard deviation; *STAI* State Trait Anxiety Inventory

We also investigated patients’ pain, obstetric and neonatal characteristics as shown in Table 3. Being multiparous (OR 0.52, 95% CI 0.30–0.90, p = 0.0202) and “sometimes” having nausea during menstruation before current pregnancy (OR 0.46, 95% CI 0.22–0.97, p = 0.0404) was significantly associated with reduced risk of having high acute postpartum pain in univariate analysis. On the other hand, “always” having nausea during menstruation before current pregnancy (OR 2.80, 95% CI 1.10–7.11, p = 0.0309), use of pethidine for labor analgesia (OR 1.61, 95% CI 1.02–2.56, p = 0.0412), underwent instrumental delivery (OR 1.83, 95% Cl 1.08–3.08, p = 0.0237), longer duration of the second stage of labor (OR 1.004, 95% Cl 1.001–1.01, p = 0.0225), increased blood loss during delivery (OR 1.01, 95% Cl 1.00–1.02, p = 0.0161), increased placental weight (OR 1.002, 95% CI 1.001–1.004, p = 0.0030), increased infant weight (OR 1.001, 95% CI 1.00–1.001, p = 0.0047), increased infant length (OR 1.13, 95% CI 1.02–1.25, p = 0.0235) and presence of shoulder dystocia (OR 6.45, 95% CI 1.59–26.16, p = 0.0090) showed a higher risk of having high acute postpartum pain at 0 to 24 h after delivery.

**Table 3:** Pain, obstetric and neonatal characteristics

Variables	Low acute postpartum pain (NRS 0–2) N = 759	High acute postpartum pain (NRS ≥ 3) N = 121	Unadjusted OR (95% CI)	P-value
Gravida, mean (SD)	1.8 (1.2)	1.9 (1.3)	1.04 (0.89–1.21)	0.6583
Gestational age (weeks), mean (SD)	38.1 (1.7)	38.1 (1.4)	1.00 (0.83–1.21)	0.9883
Parity, n (%)				
Nulliparous	394 (70.1)	77 (81.9)	Reference	--
Multiparous	168 (29.9)	17 (18.1)	0.52 (0.30–0.90)	0.0202
Menstrual cycles, n (%)				
Regular	561 (79.5)	96 (87.3)	Reference	--
Irregular	145 (20.5)	14 (12.7)	0.56 (0.31–1.02)	0.0572
Nausea during menstruation before current pregnancy, n (%)				0.0082^a^
Never	345 (49.0)	54 (47.4)	Reference	--
Rarely	219 (31.1)	44 (38.6)	1.28 (0.83–1.98)	0.2579
Sometimes	124 (17.6)	9 (7.9)	0.46 (0.22–0.97)	0.0404
Always	16 (2.3)	7 (6.1)	2.80 (1.10–7.11)	0.0309
Contraceptive use, n (%)				0.3664^a^
None	603 (91.1)	98 (92.5)	Reference	--
Oral contraceptive	15 (2.3)	4 (3.8)	1.64 (0.53–5.05)	0.3876
Others	44 (6.6)	4 (3.8)	0.56 (0.20–1.59)	0.2762
Current pregnancy, n (%)				0.4593^a^
Unplanned	195 (27.2)	27 (23.7)	0.80 (0.50–1.27)	0.3433
Planned: Natural	455 (63.5)	79 (69.3)	Reference	--
Planned: Assisted	67 (9.3)	8 (7.0)	0.69 (0.32–1.49)	0.3416
Labor analgesia, n (%)				
None	26 (3.4)	3 (2.5)	0.72 (0.21–2.41)	0.5903
Epidural analgesia	606 (79.8)	97 (80.2)	1.02 (0.63–1.65)	0.9346
Pethidine	124 (16.3)	29 (24.0)	1.61 (1.02–2.56)	0.0412
Entonox	477 (62.8)	79 (65.3)	1.11 (0.74–1.66)	0.6049
Remifentanil	8 (1.1)	1 (0.8)	0.78 (0.10–6.31)	0.8178
Mode of delivery, n (%)				0.0627 ^a^
Normal vaginal delivery	492 (64.8)	72 (59.5)	Reference	--
Emergency cesarean delivery	181 (23.8)	26 (21.5)	0.99 (0.61–1.59)	0.9395
Instrumental delivery	86 (11.3)	23 (19.0)	1.83 (1.08–3.08)	0.0237
Menstrual cycles, n (%)				
Regular	561 (79.5)	96 (87.3)	Reference	--
Irregular	145 (20.5)	14 (12.7)	0.56 (0.31–1.02)	0.0572
Postpartum obstetric complications, n (%)^b^				
None	623 (83.6)	91 (78.4)	Reference	--
Yes	122 (16.4)	25 (21.6)	1.40 (0.87–2.28)	0.1694
Neonatal complications, n (%)^c^				
None	708 (95.3)	110 (94.8)	Reference	--
Yes	35 (4.7)	6 (5.2)	1.10 (0.45–2.68)	0.8283
Duration of second stage of labor (mins), mean (SD)	62.9 (59.7)	78.3 (62.1)	1.004 (1.001–1.01)	0.0225
Blood loss during delivery (mL), mean (SD)	273.1 (135.6)	310.1 (211)	1.01 (1.00–1.02)^d^	0.0161
Placental weight (g), mean (SD)	608.5 (119.1)	643.3 (115.4)	1.002 (1.001–1.004)	0.0030
Infant weight (g), mean (SD)	3178.2 (361.4)	3279 (382.1)	1.001 (1.00–1.001)	0.0047
Infant length (cm), mean (SD)	49.2 (1.9)	49.6 (1.8)	1.13 (1.02–1.25)	0.0235
APGAR 1’ (0–10), mean (SD)	8.9 (0.6)	9.0 (0.2)	1.47 (0.77–2.80)	0.2383
APGAR 5’ (0–10), mean (SD)	9.0 (0.4)	9.0 (0.1)	1.45 (0.52–4.04)	0.4757
Shoulder dystocia, n (%)				
No	755 (99.5)	117 (96.7)	Reference	--
Yes	4 (0.5)	4 (3.3)	6.45 (1.59–26.16)	0.0090

The missing data are as follows: parity (n = 224), menstrual cycles (n = 64), nausea (n = 62), contraceptive use (n = 112), current pregnancy (n = 49), labor analgesia (n = 40), postpartum obstetric complications (n = 19), neonatal complications (n = 21), duration of second stage (n = 205), blood loss during delivery (n = 2), placental weight (n = 1), and infant length (n = 5)

*CI* confidence interval; *NRS* numerical rating scale; *OR* odds ratio; *SD* standard deviation

^a^ Type III p-value

^b^ The subcategories of postpartum obstetric complications were: prolonged labor (n = 63), emergency/crash caesarean delivery (n = 79), failed anesthesia for caesarean section (n = 1), postpartum hemorrhage (n = 5) and others (n = 24)

^c^ The subcategories of neonatal complications were: neonatal infection (n = 2), neonatal reflux (n = 6), hospitalization (n = 11), surgical conditions (n = 2), and others (n = 26)

^d^ Calculated for every 10ml blood loss

The univariate factors selected for multivariable analysis (p < 0.15) are presented in Table 4. Independent association factors for acute postpartum pain were identified: greater A-LPQ subscale on birthing pain (adjusted OR (aOR) 1.03, 95% CI 1.01–1.05, p = 0.0008), increased blood loss during delivery (for every 10ml change; aOR 1.01, 95% CI 1.00–1.03, p = 0.0148), presence of shoulder dystocia (aOR 10.06, 95% CI 2.28–44.36, p = 0.0023), and use of pethidine for labor analgesia (aOR 1.74, 95% CI 1.07–2.84, p = 0.0271). Compared with “never” or “rarely having”, “sometimes” having nausea during menstruation before current pregnancy (aOR 0.34, 95% CI 0.16–0.72, p = 0.0045) was found to be independently associated with reduced risk of high acute postpartum pain. The area under the curve (AUC) for the multivariable model was 0.66 (95% CI 0.61–0.71) (Table 4).

**Table 4:** Multivariable logistic regression model on associated factors with acute postpartum pain

Variables	Adjusted OR (95% CI)	P - value
A-LPQ birthing pain	1.03 (1.01–1.05)	0.0008
Blood loss during delivery (for every 10ml change)	1.01 (1.00–1.03)	0.0148
Presence of shoulder dystocia	10.06 (2.28–44.36)	0.0023
Use of pethidine for labor analgesia	1.74 (1.07–2.84)	0.0271
Nausea during menstruation before current pregnancy (Reference: Never/ rarely)		0.0022^a^
Sometimes	0.34 (0.16–0.72)	0.0045
Always	2.42 (0.94–6.22)	0.0666

* A-LPQ* Angle Labor Pain Questionnaire; *CI* confidence interval; *OR* odds ratio

^a^ Type III p-value

## Discussion

In this study, we found that labor pain intensity as assessed via A-LPQ total score was not associated with acute postpartum pain. However, greater A-LPQ subscale on birthing pain, increased blood loss during delivery, presence of shoulder dystocia, and use of pethidine for labor analgesia were independently associated with high acute postpartum pain in women who underwent labor process. In addition, “sometimes” having nausea during menstruation before current pregnancy was also found to be independently associated with reduced risk of high acute postpartum pain.

In the primary aim of the study, we found no association between overall labor pain intensity and acute postpartum pain; but looking into specific birthing pain revealed a significant association with high acute postpartum pain at 0 to 24 h after delivery. This may attribute to the timing of A-LPQ administration, where participants were given the choice to complete the A-LPQ at any time point after they experienced labor pain. The use of different labor analgesia and the retrospective completion of A-LPQ may further contribute to a subjective recollection of pain experience emotions and psychological state that could reduce the true representation of labor experience. In addition, other physiological domains (back pain/long haul, uterine contraction) did not show significant association with acute postpartum pain. It is notable that uterine contraction and low back pain are typically present during the first stage of labor, whereas perineal pain usually exists at the later second stage of labor [20]. After the delivery, uterine contraction pain is common within 48 h of delivery, while low back pain only persisted in 13.4% of a study cohort [18]. Thus, it is possible that uterine contraction and low back pain experiences are temporary and may not be a significant source to govern labor pain experience.

The association factors on presence of shoulder dystocia and increased blood loss during delivery may imply presence of perineal trauma or complicated birth experience in those having high acute postpartum pain. As one of the most common sources of postpartum pain, perineal pain typically arises from the stretching and tearing of perineal tissues (perineal trauma) during the period from complete cervical dilation to the delivery [21]. Several risk factors have been shown to be associated with perineal trauma, such as prolonged second stage of labor, instrumental delivery, decreased parity, larger infant birth weight, and shoulder dystocia [22, 23]. Similarly, a greater degree of perineal trauma may lead to increased blood loss during delivery, hence leading to maternal and neonatal complications and poorer postpartum recovery [24]. Our findings are in accordance with previous report that an increased degree of perineal and vaginal trauma as caused by shoulder dystocia could be associated with greater acute postpartum pain scores [22]. This highlights the importance of the early identification of individuals at risk of perineal pain and obstetric complications to allow earlier access to acute postpartum pain management.

We also identified the use of pethidine for labor analgesia as one of the association factors of high acute postpartum pain. Intramuscular pethidine is a commonly used opioid for labor pain relief; yet many studies have reported concerns regarding its use including the various side effects on nausea, vomiting, sedation, and respiratory depression [25, 26]. Studies have also concluded that pethidine is less efficacious in managing labor pain as well as acute postoperative and postpartum pain as compared with epidural and other analgesia [26, 27]. Notably, the use of pethidine during labor was independently associated with SAPC, implying that the use of pethidine during labor plays a similar role in the development of acute postpartum pain, which extends to experiencing SAPC [12, 28].

As part of the primary study assessment for perinatal mental health, we investigated the factors that could be associated with postpartum depression, which included symptoms in premenstrual syndrome (PMS) which has been reported to have positive association with postpartum depression [29]. “Sometimes” having nausea during menstruation before current pregnancy was found to be a protective factor against high acute postpartum pain as compared with those who “never” or “rarely” had nausea. The univariate analysis showed a reverse trend on this factor, such that “always” having nausea during menstruation before current pregnancy contributes to high acute postpartum pain. This may be attributed to other factors not analyzed in this study that may have mediated the association between nausea and acute postpartum pain. A plausible explanation could be the effects brought by hormonal changes, as evidence has shown that nausea during the menstrual cycle and pregnancy are linked to reproductive hormones (e.g., estrogen, progesterone) [30, 31]. Furthermore, having low estrogen levels may exacerbate pain, while a consistent mid-range or high level of estrogen may reduce pain in premenopausal women [32]. Future studies are needed to unravel the exact relationship between the gonadal hormones, nausea, and pain experience.

It was previously reported that pain catastrophizing may predict labor pain intensity and anticipation, leading to a greater risk of acute and persistent perineal postpartum pain and poorer postpartum maternal recovery and adjustment [3, 33, 34]. Similarly, fear-avoidance was shown to be a predictor of intrapartum pelvic girdle pain, genito-pelvic and lumbopelvic pain at 3- and 6-months postpartum [5, 10, 11]. Nonetheless, we did not find any significant association between pre-delivery pain catastrophizing and fear-avoidance with acute postpartum pain in this study. An explanation for our results could be the role of pain-related acceptance within the fear-avoidance model. Pain-related acceptance is the ability and willingness to experience pain, and is found to mediate the associations between pain, pain catastrophizing, and fear-avoidance beliefs [35, 36]. Previous study suggests that a higher level of pain-related acceptance may be protective against the development of fear-full catastrophic beliefs of pain and the maladaptive cognitive-behavioral cycle described in the model [36]. Thus, more research is needed to delineate the role of pain-related acceptance in childbirth and its effects on pain catastrophizing and fear-avoidance beliefs.

This study should consider several limitations. First, this study was conducted in a single maternity institution that offers treatment to predominantly English-speaking Asian population. A systematic review showed that Asians could have lower pain thresholds and different perceptions towards pain as compared with other populations, e.g., non-Hispanic whites, hence limiting the extrapolation of the study findings to other populations [37]. Secondly, other potential factors (religion, income, and socioeconomic status, postpartum analgesia (e.g., paracetamol, mefenamic acid, tramadol)) confounding to labor pain intensity and acute postpartum pain were not investigated in this study [38, 39]. Thirdly, as this was intended to be a secondary analysis, we did not include the sample size calculation in the manuscript. We did perform a post-hoc power calculation as below: Based on available sample size of 880 (121 in high and 759 in low acute postpartum pain groups) study has at least 80% power to reject the null hypothesis of conservative mean difference (Δ) of 14 points in mean A-LPQ scores between high and low acute postpartum pain groups with a common SD σ of 43 based on two-sided independent equal-variance t-test. The targeted A-LPQ score difference of Δ = 14 points is considered to be clinically meaningful and translates to a small effect size of Δ/σ = 14/43 = 0.33 [40]. Finally, the collected data largely rely on patient self-reporting especially the administered questionnaires before the delivery. Given the dynamic and subjective nature of the labor experience, the measurement of labor pain is particularly challenging as the use of self-reporting questionnaires may lead to recall bias or inconsistent results. Additionally, the lack of strict standardization for the exact timing of questionnaire administration may further affect the results as the participants may be in different physical or emotional states at the time of questionnaire response. We acknowledge this limitation, but we allowed women to provide their responses retrospectively for study compliance with reduced participation stress during painful laboring periods.

## Conclusion

In summary, pre-delivery pain factor together with obstetric complications (shoulder dystocia, blood loss during delivery) were independently associated with high acute postpartum pain. Future studies are warranted to improve the performance of the model that could help to define the population at risk of high acute postpartum pain. Identification of modifiable pain and psychological factors could also enable healthcare professionals to implement pre-emptive interventions to improve perinatal and postpartum pain management.

## Data Availability

The datasets generated and/or analyzed during this study are not publicly available due to institutional policy on data confidentiality but are available from the corresponding author on reasonable request.

## Abbreviations

A-LPQ: Angle Labor Pain Questionnaire
aOR: adjusted odds ratio
ASA: American Society of Anesthesiologists
BMI: body mass index
CI: confidence interval
FACS: Fear Avoidance Components Scale
NRS: Numeric Rating Scale
PCS: Pain Catastrophizing Scale
PMS: premenstrual syndrome
SAPC: sub-acute pain after childbirth
SD: standard deviation
STROBE: Strengthening the Reporting of Observational Studies in Epidemiology
VAS: Visual Analog Scale
VRS: Verbal Rating Scale
